# Processing, Mechanical and Morphological Properties of GTR Modified by SBS Copolymers

**DOI:** 10.3390/ma16051788

**Published:** 2023-02-22

**Authors:** Agnieszka Susik, Agata Rodak, Javier Cañavate, Xavier Colom, Shifeng Wang, Krzysztof Formela

**Affiliations:** 1 Department of Polymer Technology, Faculty of Chemistry, Gdańsk University of Technology, Gabriela Narutowicza 11/12, 80-233 Gdańsk, Poland; 2 Advanced Materials Center, Gdańsk University of Technology, Gabriela Narutowicza 11/12, 80-233 Gdańsk, Poland; 3 Department of Chemical Engineering, Universitat Politècnica de Catalunya Barcelona Tech, Colom 1, Terrassa, 08222 Barcelona, Spain; 4 Department of Polymer Science and Engineering, Shanghai State Key Lab of Electrical Insulation and Thermal Aging, Shanghai Jiao Tong University, Shanghai 200240, China

**Keywords:** ground tire rubber, SBS copolymers, modification, melt-blending, recycling

## Abstract

In this work, ground tire rubber (GTR) was thermo-mechanically treated in the presence of styrene-butadiene-styrene (SBS) copolymers. During preliminary investigation, the effects of different SBS copolymer grades, the variable content of SBS copolymer on the Mooney viscosity, and the thermal and mechanical properties of modified GTR were determined. Subsequently, GTR modified by SBS copolymer and cross-linking agents (sulfur-based system and dicumyl peroxide) was characterized by assessment of rheological, physico-mechanical, and morphological properties. Rheological investigations showed that linear SBS copolymer, with the highest melt flow rate among studied SBS grades, was the most promising modifier of GTR, considering processing behavior. It was also observed that an SBS improves the thermal stability of the modified GTR. However, it was found that higher content of SBS copolymer (above 30 wt%) does not bring any effective changes and, for economic reasons, is inefficient. The results showed that samples based on GTR modified by SBS and dicumyl peroxide have better processability and slightly higher mechanical properties compared to samples cross-linked by a sulfur-based system. This is due to the affinity of dicumyl peroxide to the co-cross-linking of GTR and SBS phases.

## 1. Introduction

The development of the automotive industry is strongly affecting the level of global production of rubber-based materials. According to statistics provided by European Tyre and Rubber Manufacturers’ Association (ETRMA), the automotive sector accounts for 65% of the production of general rubber goods [1]. Generally, rubber-based materials used in the construction of vehicles can be categorized into five main streams, including 67 wt% of tires, 9 wt% of weather strips, 8 wt% of vibration isolators, 6 wt% of hoses, and 10 wt% of other products [2]. This classification clearly indicates that waste tires constitute the main stream of waste rubber from the automotive industry. Considering the present demand for new tires, the efficient management and recycling of waste tires will be increasingly necessary. For example, in 2020, in Europe alone, the sales of new tires reached 324 million units, which includes 70 wt% for passenger cars and light-duty vehicles, 20 wt% for heavy-duty vehicles (trucks and busses), 1 wt% for motorbikes and scooters, and 9 wt% for agricultural and off-road vehicles [3]. Moreover, current trends show that in 2030 the number of waste tires generated annually to the environment can reach 1.2 billion [4]. This means an increase of around 20% compared to present levels.

In this situation, the lack of strategies for properly recycling waste tires is a serious threat to the environment and human health. An added problem is that the illegal landfilling of waste tires increases the possibility of spontaneous combustion. Although waste tires are not easy to ignite, their fires are very difficult to extinguish due to the high calorific value of tires, around 30–40 MJ/kg, which is higher than those of coal and other solid fuels [5]. Moreover, the accumulated tires’ large volume of void space supplies the air to the fire [6].

The accumulation of waste tires also increases the risk of cancer for people living near illegal waste tire stocks because, during their combustion, toxic, mutagenic, and carcinogenic compounds (e.g., heavy metals, PAHs, volatile organic compounds, pyrolytic char or oil, etc.) are emitted to the environment [7,8,9].

Estimated data show that in the European Union countries and the United Kingdom in 2019, 3,164,282 tons of waste tires were engendered to the environment, and almost 95% were collected and treated for material recycling (1,764,725 tons) and energy recovery (1,254,796 tons) [10], while for only around 5% (136.279 tons) the utilization method is unknown (it is most likely that waste tires were illegally dumped). This data clearly shows that the level of management and further treatment of waste tires in the European Union and the United Kingdom is very high, and it seems that the effectiveness of waste tire recycling should increase in the near future.

However, it should be mentioned that the presented data are average values, and such a high level of recycling is not equally distributed in Europe. For example, consider two countries with similar levels of waste tires generated each year: Spain (238,080 tons) and Poland (268,500 tons). In Spain, the treated waste tire level is 100%, while in Poland, it is only 79%. Moreover, considering the waste tires utilized by unknown methods in European Union countries and the United Kingdom, surprisingly, almost 42.2% (57,500 tons) were located in Poland. This problem can be partially resolved by properly selecting the locations for recycling plants and the number of waste tires collection points [11]. In addition, legislative frameworks and circular economy strategies, such as the 4R strategy (reduce, reuse, recycle, and recover) [12] or the more recent 7R model (reduce, reuse, recycle, redesign, renew, repair, and recover) [13], partially enforce the industry to invest in research and development of the projects related to the rubber recycling technologies.

At present, the main method of waste tire recycling is based on their mechanical disintegration by shredding, grinding, and pulverization in order to obtain ground tire rubber (GTR) [14,15,16]. GTR with suitable particle size and distribution is high-quality (when properly sorted and cleaned) starting material for further recycling of waste tires. Many applications of untreated, reclaimed, or modified/functionalized GTR, mostly as modifiers or low-cost filler in different materials, have been considered so far [17,18,19]. However, in many cases, promising laboratory-scale results are difficult to implement at an industrial scale. The main problems in upscaling are related to complex protocols used in rubber recycling (e.g., multi-steps of material preparation, purification of obtained products, etc.), material and energetic costs of waste rubber treatment (e.g., high-temperature processes, long-time of treatment, use of expensive or unavailable additives, etc.), and also environmental aspects (e.g., emission of volatile degradation products, use of toxic chemicals, etc.).

Therefore, in the field of rubber recycling, very promising methods for GTR-based materials preparation are melt-compounding and reactive extrusion [20,21]. This approach allows functionalization or modification of GTR, which is usually combined with rubber degradation, devulcanization (selective scission of cross-linking bonds), or a reclaiming process (combination of rubber degradation and cross-linking bonds). The efficiency of waste rubber thermo-mechanical treatment and the selectivity of the process are strictly related to extrusion parameters [22,23,24].

Main chain scission and selective degradation of the three-dimensional network present in cross-linked waste rubbers significantly improve their processing/flowability, increasing the waste rubbers’ level in the new rubber goods. This rubber recycling method fits the concept of sustainable development [25], which is beneficial for both environmental and economic reasons.

The literature data shows that a relatively small amount of thermoplastic polymers 10–25 wt%) act like plasticizers or binders during GTR reclaiming [26], which improves the thermal stabilization of extrusion and results in a reclaimed GTR with higher content sol fraction (which implies better processing) and/or enhanced performance properties. In this field, the most popular additive is trans-polyoctenamer (tradename: Vestenamer^®^ 8012), which is dedicated to rubber recycling [27,28]. However, other commercially available and more common thermoplastics, such as polyethylene, polypropylene or ethylene-vinyl copolymers, were also used as additives during GTR treatments [29,30,31]. Furthermore, thermoplastic elastomers can also be considered additives during the thermo-mechanical devulcanization of GTR [32,33], especially when such materials have huge potential to be applied in thermoplastic elastomers [34,35] or as asphalt modifiers [36,37]. However, the data published in this field so far are rather very limited.

Therefore, in this work, GTR was thermo-mechanically treated in the presence of styrene-butadiene-styrene (SBS) copolymers. The effects of different SBS copolymer grades (three linear and one radial), the variable content of SBS copolymer on the Mooney viscosity, and the thermal and mechanical properties of modified GTR were investigated. Moreover, for selected systems, the effect of cross-linking agents, a sulfur-based system, or dicumyl peroxide, was also determined to check and verify how cross-linking efficiency affects the processing, performance properties, and microstructure of GTR modified by SBS copolymers.

## 2. Materials and Methods

### 2.1. Materials

Ground tire rubber (GTR) obtained from a mix of passenger and truck tires, with particle sizes up to 0.6 mm, was received from Grupa Recykl S.A. (Śrem, Poland). The basic components of GTR are natural rubber (NR), styrene-butadiene rubber (SBR), butadiene rubber (BR), additives (curing system, activators, plasticizers, etc.), carbon black, silica, and ash. GTR composition determined by thermogravimetric analysis was rubbers and additives (63.1 wt%), carbon black (28.4 wt%), and ash content (8.5 wt%).

Four grades of SBS copolymers, three linear and one radical produced by Sibur Holding (Moscow, Russia), were purchased from Konimpex Sp. z o.o. (Konin, Poland). According to information from the technical data sheet, linear styrene-butadiene thermoplastic elastomers (SBS L 7322; SBS L 7342, and SBS L 7417) and radial styrene-butadiene thermoplastic elastomer (SBS R 7382) are essentially a product of styrene and butadiene block polymerization in hydrocarbon solution in the presence of an alkyllithium catalyst. The content of bonded styrene in the used SBS copolymers was: 27.5–30.5 wt% for SBS L7322, 28.5–31.5 wt% for SBS L7342, 36.0–38.0 wt% for SBS L 7417, and 28.5–31.5 wt% SBS R 7382. All studied SBS copolymers were dusted with calcium stearate, zinc stearate, or amorphous silica. The materials contain a no-staining stabilizer. Minimal values of tensile strength and elongation at break declared by the producer in the technical data sheet are in the range of 1.7–14.7 MPa and 250–800%, respectively.

Dicumyl peroxide (DCP) was supplied by Pergan GmbH (Bocholt, Germany).

The sulfur-based curing system, including stearic acid, zinc oxide, 2-mercaptobenzothiazole (MBT) accelerator, N-tert-butyl-2-benzothiazolesulfenamide (TBBS), and sulfur was provided from Standard Sp. z o.o. (Lublin, Poland).

### 2.2. Sample Preparation

#### 2.2.1. GTR Thermo-Mechanically Treated in the Presence of SBS Copolymers

Thermo-mechanical treatment of GTR in the presence of SBS copolymers was performed using Brabender^®^ internal mixer (type GMF 106/2) (Brabender GmbH & Co. KG, Duisburg, Germany) with a chamber volume of approx. 55 cm^3^, equipped with roller-type rotors. The fill factor of the mixer was 0.7, and the rotor speed was 100 rpm.

The mixing temperature and time were 200 °C and 8 min, respectively. The compression was performed under a pressure of 10 MPa. Subsequently, GTR treated by SBS copolymers was compressed at 200 °C into 2 mm-thick tiles and, before demolding, was cooled down to room temperature. Prepared samples were coded according to the used SBS copolymer grade and content, e.g., GTR + 10 wt% SBS 7322.

#### 2.2.2. GTR Modified by SBS and Curing System

In order to improve the mechanical properties of studied composites, selected materials prepared according to the protocol presented in Section 2.2.1 were mixed with a suitable curing system, the sulfur-based system (composition in parts per hundred rubber (phr): ZnO—2.5; stearic acid—0.3; TBBS—0.9; MBT—0.9; sulfur—1.5), or DCP (2 phr) using a laboratory two-roll mills 200 × 400 mm from Buzuluk Komarov. It is well known that cross-linking agents have a significant impact on the cross-link density, microstructure, and performance properties of prepared materials. Sulfur vulcanization is a complex and intricate process, which leads to the formulation of sulfidic cross-links with various lengths (mono-, di- and poly-sulfidic bonds), while peroxide curing is simplest and results in the formulation of carbon–carbon bonds. Generally, rubbers cured by sulfur possess high tensile strength, and good elastic and dynamic properties [38], while rubbers cross-linked by peroxide have good electrical properties and high temperature aging resistance [39]. More details about the advantages and disadvantages related to the application of sulfur- and peroxide-based systems in rubber processing were comprehensively described by Kruželák et al. [40].

In this study, for clarity, samples that include a curing system were additionally coded at the end of their name by “–S” in the case of the sulfur curing system and “–DCP” for dicumyl peroxide.

Subsequently, GTR samples treated by SBS and a curing system were cured at 170 °C into 2 mm-thick tiles under the pressure of 10 MPa and remolded immediately after curing. Samples were cured according to the determined optimal curing time (t_90_), defined as the time when 90% of maximal torque is reached.

### 2.3. Methodology

Fourier transform infrared spectroscopy (FTIR) analysis of used materials was performed using an IRtracer 100 from Shimadzu (Kyoto, Japan). The device was equipped with a single-reflection ATR accessory with a prism made from germanium crystal. Measurements were performed in an attenuated total reflectance mode at 4 cm^−1^ resolution and 45 scans in the range 4000–500 cm^−1^.

The melt flow rate (MFR) of SBS copolymers and GTR/SBS blends were determined at 200 °C, with a load in the 5–21.6 kg range according to ISO 1133, using Mflow plastometer from Zwick (Ulm, Germany). A wide range of loads was used to check the possibility of material flowability at variable pressure. 

The Mooney viscosity of the rubber compounds was measured at 100 °C using a Mooney viscometer MV2000 (Alpha Technologies, Akron, OH, USA) according to ISO 289-1.

The vulcanization process was investigated and recorded via Premier RPA Alpha Technologies (Hudson, OH, USA) according to the ISO 6502 standard. Further calculations were made in order to determine characteristic values for curing curves. The cure rate index (CRI), which shows the cross-linking rate, was calculated according to Equation (1):

(1) $$\mathrm{CRI}=\frac{100}{t_{90}-t_{2}}$$

where *t*_90_: optimum cure time, min; *t*_2_: scorch time, min.

The tensile strength and elongation at break were estimated in accordance with ISO 37. Tensile tests were performed on a Zwick Z020 apparatus (Ulm, Germany) at a constant speed of 200 mm/min. Shore hardness type A was estimated using a Zwick 3130 durometer (Ulm, Germany) in accordance with ISO 7619-1. At least 5 measurements per sample were performed.

Dynamic mechanical analysis was performed using the DMA Q800 TA Instruments apparatus (New Castle, DE, USA). Samples cut to the dimensions of 40 × 10 × 2 mm were loaded with a variable sinusoidal deformation force in the single cantilever bending mode at the frequency of 1 Hz under the temperature rising rate of 4 °C/min within the temperature range between −80 and 60 °C.

The density of samples was measured based on the Archimedes method, as described in ISO 1183. Accordingly, all measurements were carried out at room temperature in a methanol medium. At least 3 measurements were performed per sample.

The surface morphology created by breaking the samples in the tensile test at a 200 mm/min speed was observed with a JEOL 5610 scanning electron microscope (Tokyo, Japan) with a low-vacuum secondary electron detector. During the analysis, the electron beam accelerating voltage was 10 kV. Before measurement, the samples were covered with a fine gold layer in order to increase their conductivity in the vacuum chamber.

Thermogravimetric analysis (TGA) was performed on two apparatuses. Uncured GTR/SBS samples were measured using a Netzsch TG 209 (Selb, Germany) apparatus using samples of approximately 10 mg in the temperature range of 25–800 °C and under a nitrogen atmosphere at a heating rate of 10 °C/min. Cross-linked GTR/SBS samples were investigated using a Mettler Toledo TGA/SDTA 851 apparatus (Columbus, OH, USA). Samples weighing approximately 10 mg were placed in a corundum dish. The measurement was conducted in the temperature range of 25–800 °C and under an air atmosphere at a heating rate of 20 °C/min.

## 3. Results and Discussion

### 3.1. Chemical Structure of GTR and SBS Copolymers

**Figure 1:**
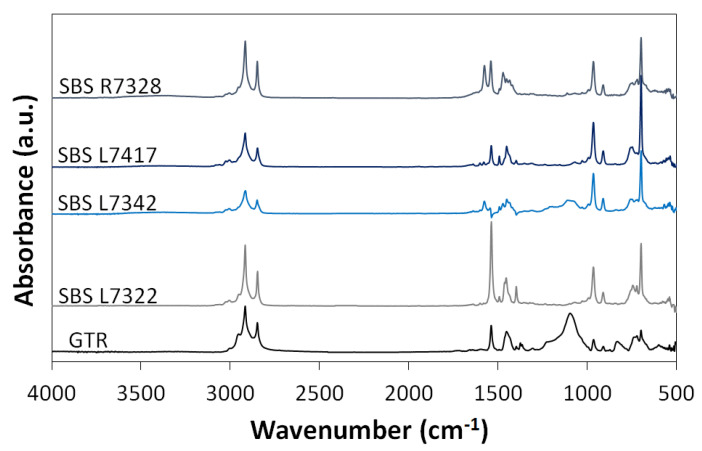
FTIR analysis of GTR and SBS copolymers.

The chemical structure of individual SBS copolymers and GTR were analyzed by FTIR analysis, and the obtained results are presented in Figure 1. As can be noticed, the chemical structure of both used materials: GTR and SBS copolymers are very similar. The C-H bond bands of the CH_2_ groups present in the aliphatic chains occur at 2916 cm^−1^ and 2850 cm^−1^ presence in both GTR and SBS. The absorbance maxima at 1540 cm^−1^ is related to the presence of C=C stretching vibrations [41]. The peak at 966 cm^−1^ is related to the presence of a dominant 1,4-trans-polybutadiene and represents the C-H bending band of the C=C bonds, while the peak at 699 cm^−1^ is due to the mono-substituted benzene rings in the polystyrene chain [42]. All of these peaks are of similar intensity, except for the peak at 699 cm^−1^ for SBS L7417. This peak is more intense than for the other copolymers, indicating a higher styrene content in this type of SBS. According to the producer data sheet, for SBS L7417, bound styrene content is 36–38 wt%, while other SBS copolymers used in this paper were in the range of 27.5–31.5 wt%. The main difference in the analyzed FTIR spectra can be noticed for maximum absorbance at 1375 cm^−1^ and 831 cm^−1^, which was detected only in the case of the GTR. The peak at 1375 cm^−1^ is attributed to the scissoring vibration of CH_3_, while the signal at 831 cm^−1^ indicates out-of-plane bending vibration of C–H in the –CH=CH– group of cis-1,4-unit. Both signals are characteristic of natural rubber [43,44], the presence of which was also confirmed by thermogravimetric analysis of GTR.

### 3.2. MFR of SBS Copolymers and GTR Modified by SBS Copolymers

The obtained MFR results and the appearance of studied materials after the test are presented in Table 1. It should be mentioned that a standardized capillary die with a diameter of 2.095 mm and a height of 8 mm for all melt flow rate studies was used. Additionally, a non-standardized capillary with higher diameter 4.190 mm and height 8 mm was also applied for composition characterized by poor flowability. As can be observed, although different MFR measurement conditions were used (variable load and capillary die diameter), only for pure SBS L3422 and SBS L7417 copolymers was it possible to determine MFR at 200 °C, while MFR for GTR modified by SBS copolymers was unmeasurable in all measurement conditions. Garcia et al. [45] showed that a high-pressure capillary rheometer and shear rate between 300 and 15,000 s^–1^ is a much better method for this paper. The authors’ results indicated that the sol fraction of studied devulcanized GTR should be at least 27%.

### 3.3. Mooney Viscosity and Physico-Mechanical Properties of GTR Modified by SBS Copolymers

**Figure 2:**
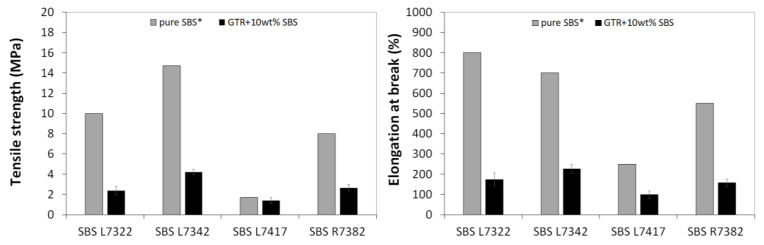
Tensile strength and elongation at break of pure SBS copolymers (* data from technical data sheet provided by producer) and GTR modified by 10 wt% of SBS copolymer as a function of SBS grade.

**Figure 3:**
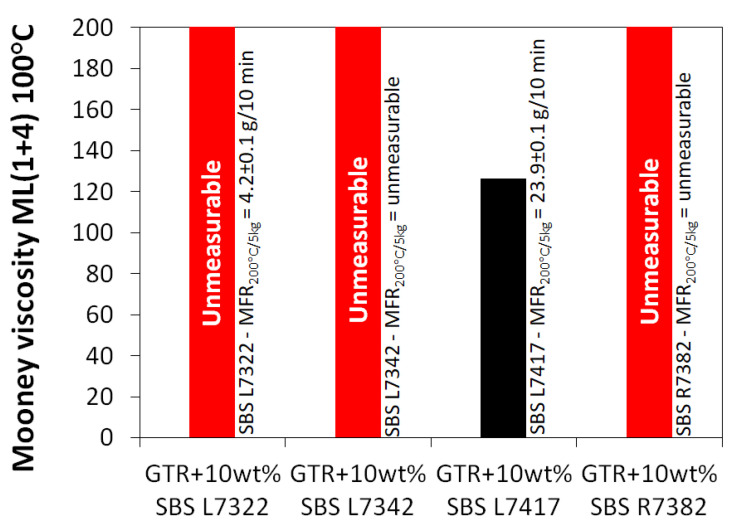
Mooney viscosity of GTR modified by 10 wt% of SBS copolymer as a function of SBS grade.

During sample preparation, we were guided by two main parameters: (i) the tensile strength and (ii) the Mooney viscosity of the studied material. Our assumption was to prepare the material with satisfied flowability, confirmed by the possibility of Mooney viscosity measurements. On the other hand, the tensile strength of prepared materials should be as high as possible with the smallest possible addition of SBS copolymer as a modifier. Figure 2 shows that the GTR + 10 wt% SBS L7342 with tensile strength 4.2 MPa showed the highest value compared to other SBS grades. The tensile strength of the GTR + 10 wt% SBS L7342 sample is about three times higher than that of GTR + 10 wt% with SBS L7417, which had the lowest tensile strength (about 1.5 MPa). Moreover, the SBS L7342 copolymer has the highest tensile strength among the studied SBS copolymers (about 15 MPa). Nevertheless, it is not possible to consider only the tensile strength results when selecting a material. It is important that the material can also be processed well at low modifier content, which is crucial for further use of such material in the rubber industry. Figure 3 shows the results of Mooney viscosity measurements for GTR modified by 10 wt% of SBS copolymer as a function of SBS grade. As observed, the Mooney viscosity could be determined only for sample GTR + 10 wt% SBS L7417, while for other samples initial point was too high to start measurement. It should also be mentioned that processing pure GTR in the same conditions resulted in a material that cannot be characterized by a Mooney viscometer.

**Figure 4:**
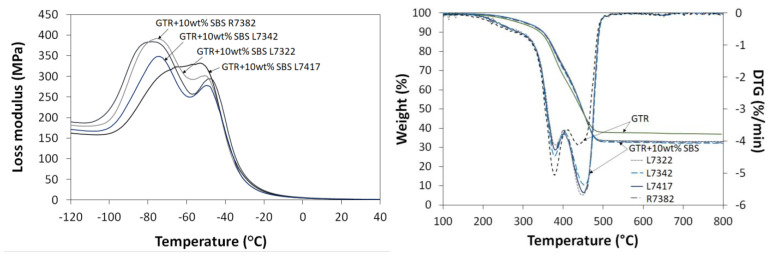
DMA and TGA results for GTR modified by 10 wt% of SBS copolymer as a function of SBS grade.

In the next step of the preliminary investigation, the studied materials were characterized by dynamic mechanical analysis (DMA), thermogravimetric analysis, and derivative thermogravimetry (TGA and DTG). The obtained results are presented in Figure 4.

DMA records the temperature-dependent viscoelastic properties of the material, where loss modulus (E’’) represent the viscous part and is correlated to the energy dissipation in the sample [46]. The dependence of loss modulus as a function of temperature allows for observing how the SBS copolymer grade affects the glass transition temperature of studied materials.

It can be observed that the peak values of the loss modulus curves are similar for GTR modified with 10 wt% of SBS R7382, SBS L7342, and SBS L7322. Two peaks on loss modulus curves can be observed; the maximum at about −70 °C is related to the glass transition temperature of SBS copolymer, and the maximum at about −50 °C corresponds to the glass transition of GTR [47,48].

On the other hand, for GTR treated by 10 wt% of SBS L7147, the loss modulus curve is significantly different for other used SBS copolymers, and only one broad peak is observed, which might be related to the highest content of bound styrene for SBS L7417 compared to other used SBS copolymers (please see Section 3.1.).

Thermogravimetric analysis is a useful analytical technique in rubber recycling products assessment, which allows for determining the thermal stability and composition of the prepared GTR-based materials [49,50]. The content of carbon black and ash in GTR was 36.9 wt%. GTR degradation showed two characteristic peaks (T_max1_ and T_max2_) on derivative thermogravimetry (DTG) curves. T_max1_ at around 390 °C is related to natural rubber and butadiene rubber degradation, while T_max2_ at around 460 °C corresponds with styrene-butadiene rubber and butadiene rubber degradation, which are rubbers commonly used in the tire industry [51].

As can be observed, regardless of SBS grade, the magnitude of T_max2_ corresponding to synthetic rubber increased, which confirms that the amount of used additive was the same in all studied materials. It was also noticed that the application of SBS copolymers improves the thermal stability of studied materials due to the higher thermal stability of SBS than GTR.

Considering the above-presented preliminary results of studied materials, during further research, we decided to focus on GTR modified with SBS L7417 copolymer, which was characterized by acceptable Mooney viscosity. At the same time, tensile strength is comparable to untreated GTR after reactive sintering, which can be in the range of ~0.6–2.2 MPa [52,53].

**Figure 5:**
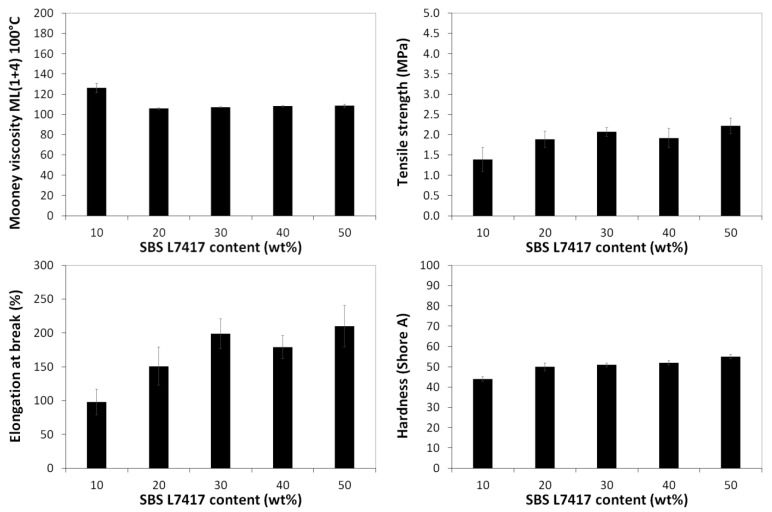
Mooney viscosity and mechanical properties of GTR modified by variable content of SBS L7417.

In order to improve the processing and tensile strength of studied samples, the simplest strategy can be to increase the used thermoplastic elastomer modifier. Therefore the Mooney viscosity and mechanical properties of GTR modified by variable content (up to 50 wt%) of SBS L7417 are summarized in Figure 5. As can be noticed, GTR + 10 wt% SBS L7417 was characterized by the highest Mooney viscosity while increasing the content of the modifier to 20 wt% resulting in the decrease of this parameter of ~20%. However, further modifier increases did not significantly change the processing of studied materials. In addition, it can be seen that at a modifier content of 10 wt% to 30 wt%, the tensile strength improves (from 1.4 to 2.1 MPa), while for the sample with modifier content above 30 wt%, the increase of tensile strength is rather limited. Similar relationships can be observed for the elongation at break. The hardness of studied materials increased with higher content of SBS L7417 copolymer as a modifier (from 44 Shore A for GTR + 10 wt% SBS L7417 to 55 Shore A for GTR + 50 wt% SBS L7417).

### 3.4. Mooney Viscosity and Curing Characteristics of GTR Modified by SBS and Curing System

As presented above, small changes in Mooney viscosity and mechanical properties show that it is pointless to use larger contents of SBS copolymer in the GTR. Therefore, to make the process more economical, the modifier was reduced to 30%. To improve the physico-mechanical properties of studies materials, further studies were focused on GTR modified by SBS L7417 and suitable curing agents: the sulfur-based system and dicumyl peroxide, respectively.

**Figure 6:**
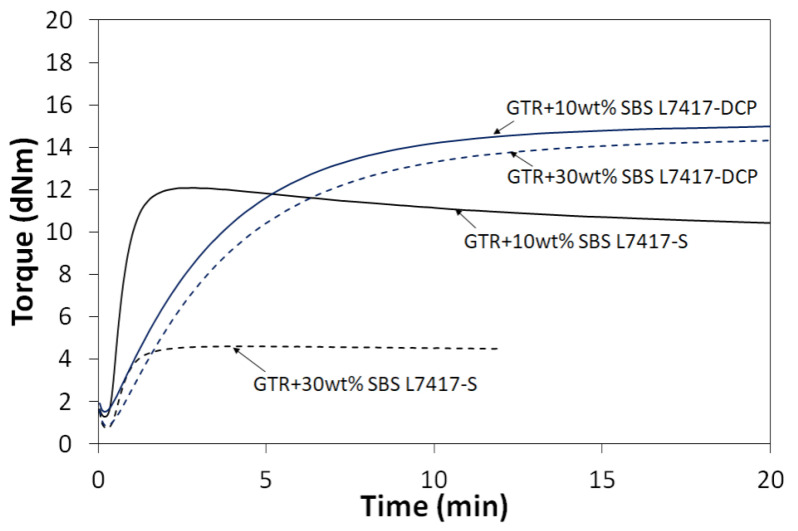
Curing curves of GTR modified by SBS and cross-linking agents.

The effect of SBS L7417 content and cross-linking system on the processing and curing behavior of the modified GTR are shown in Figure 6 and summarized in Table 2. It can be noted that the Mooney viscosity for GTR modified by SBS L7417 and the cross-linking system is lower than for GTR treated by SBS copolymer, which is due to the addition of shear forces acting on the material during the mixing of modified GTR with cross-linking agents using two-roll mills cooled by water. However, surprisingly, GTR treated by SBS and DCP Mooney viscosity is lower than in systems with sulfur, as evidenced by the decrease of the Mooney viscosity from 94.6 to 87.1 for GTR + 10 wt% SBS L7417 and from 93.8 to 77.5 for GTR + 30 wt% SBS L7417. This is due to the melting point of dicumyl peroxide, which is around 40 °C. Therefore, melted DCP can act as a plasticizer in modified GTR during Mooney viscosity measurement at 100 °C. This observation confirms that DCP did not react when blending with GTR on two roll mills.

The minimum torque (M_L_) is also a parameter that indicates the processing properties of materials. The lower its value, the better the processing properties of the sample. As expected, this parameter’s value decreases with an increasing amount of SBS copolymer. However, for samples cross-linked by dicumyl peroxide, the M_L_ parameter is slightly higher, regardless of SBS copolymer content in the modified GTR. This might be related to better cross-linked efficiency than the sulfur-based system because similar trends can be observed for maximal torque (M_H_) and torque increment (∆M) determined for studied materials. The smallest values of M_H_ and ∆M were determined for GTR + 30 wt% SBS L7417-S, which indicates the partial degradation of material during cross-linking or lower cure efficiency for this sample.

Scorch time (t_s2_) for all studied GTR-based materials was similar and very short in the 0.5–0.9 min range, and the lower values were determined for samples with a sulfur-based system. On the other hand, optimum cure time (t_90_) values showed that systems of GTR modified by SBS and DCP and cured at 170 °C need more time to form a three-dimensional network and chain stabilization than systems with sulfur. The higher content of the SBS modifier resulted in an increase in the CRI value.

Changing the proportion of the SBS modifier resulted in an increase in the CRI value, which is related to the affinity of curing agents to GTR and SBS and, consequently, a difference in the mechanism of cross-linking bond formulation and curing rate.

### 3.5. Physico-Mechanical Properties of GTR Modified by SBS and Curing System

Physico-mechanical properties modified by SBS and the curing system were summarized in Table 3. As can be noticed, the obtained materials are characterized by much higher (~4–5 times) tensile strength (in the range of 7.3–8.1 MPa) compared to uncured samples, while elongation at break (in the range of 171–217%) remains at the similar level (please see Figure 5). The tensile strength of prepared materials is higher than results obtained for commercially available reclaimed rubbers (tensile strength in the range: 4.6–5.2 MPa) [54] or thermoplastic elastomers based on low-density polyethylene/styrene-butadiene-styrene copolymer/GTR blends with maximal content of GTR ~50 wt% (tensile strength in the range 5.7–6.8 MPa) [55]. On the other hand, it can be observed that regardless of the modifier content used, the studied materials have similar tensile strength. In the case of elongation at break, it can be noticed that this parameter’s value increases with higher SBS copolymer content. A similar tendency was observed for hardness, which for studied materials increases with the higher content of the SBS copolymer as a modifier, while the effect of cross-linking agent type on this parameter was negligible. The density of the samples decreased when the amount of SBS copolymer was increased and is lower for the system with DCP. This is simply due to the specifications of the used components. DCP has a lower density (about 1.56 g/cm^3^) density than sulfur (about 2.07 g/cm^3^) or zinc oxide (5.61 g/cm^3^) [56].

### 3.6. Microstructure of GTR Modified by SBS and Curing System

**Figure 7:**
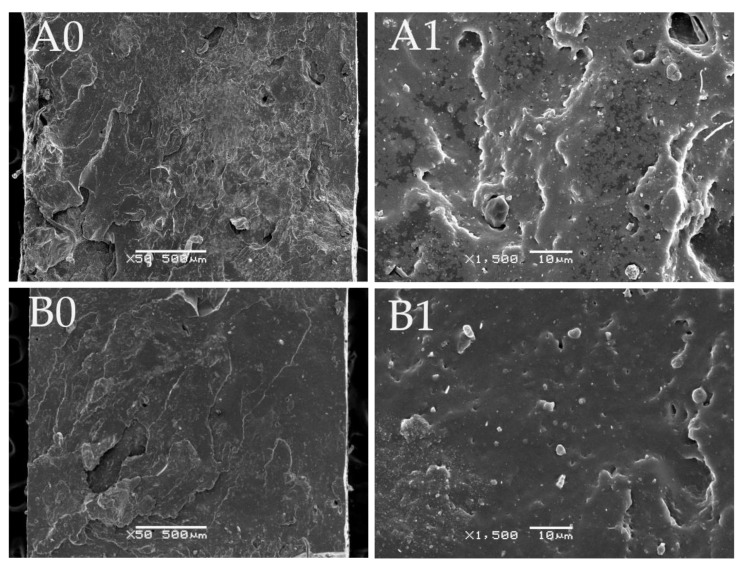
SEM images of: A0, A1—GTR + 10 wt% SBS L7417-S (magnification × 50; magnification × 1500); B0,B1— GTR + 10 wt% SBS L7417-DCP (magnification × 50; magnification × 1500).

The scanning electron microscopy (SEM) technique was used to assess the breaking mechanism of studied materials. For this purpose, SEM analysis of surface area perpendicular to the direction of strain was performed at two magnifications: x50 and x1500. SEM images for samples GTR + 10 wt% SBS L7417-S and GTR + 10 wt% SBS L7417-DCP are presented in Figure 7. It can be seen that the GTR modified with SBS and the sulfur-based system have a rough surface containing many cracks and voids, while the surface of GTR modified with SBS and DCP is smoother and better distributed. This can be related to the curing efficiency of the tested materials. As mentioned in Section 3.2., in the case of a sulfur-based system in GTR modified by SBS, the GTR phase is mainly involved in the curing process. On the other hand, DCP decomposition products allow for co-cross-linking GTR and SBR phases in GTR modified by SBS systems, which results in more homogeneous morphology. Moreover, it should be mentioned that the MBT accelerator in a sulfur-based system might enhance rubber devulcanization [57,58], which resulted in volatile compound emissions and, consequently, voids formulation in GTR+10 wt% SBS L7417-S sample.

### 3.7. Thermal Stability of GTR Modified by SBS and Curing System

**Figure 8:**
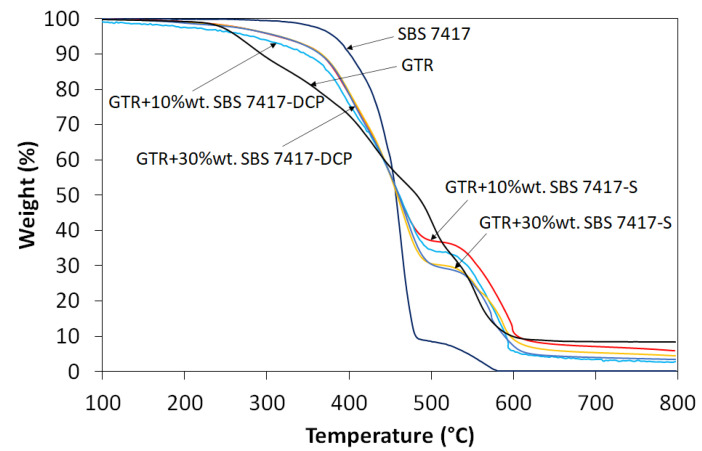
TGA curves for GTR modified by SBS and curing agents.

The thermal stability of studied materials was investigated using thermogravimetric analysis (TGA) performed in the air atmosphere, and the obtained results are presented in Figure 8 and summarized in Table 4. TGA curves show three main peaks attributed to thermal decomposition: the natural rubber and butadiene rubber present in the GTR (~400 °C), synthetic rubbers present in the GTR and SBS (~430 °C), and a broad peak corresponding to carbon black (~580 °C) [59]. For the materials tested, the type of cross-linking system has an insignificant effect on char residue formulation.

Moreover, the results showed that SBS L 7417 copolymer has higher thermal stability than studied materials, which confirms that higher content of this modifier improves the thermal stability of GTR (please see also Figure 4). It was found that T_−2%_ and T_−5%_, which corresponded to temperature with weight loss of sample 2% and 5%, were higher for GTR modified by SBS and the sulfur-based system than in the case of the sample with DCP. This tendency was especially observed for the sample with 10 wt% of SBS L 7417 copolymer. This observation can be related to two factors. The first factor is due to the thermal decomposition of dicumyl peroxide, resulting in cross-linking of material and the formulation of low molecular weight by-products [60], which have a lower degradation temperature than modified GTR. The second factor is related to partial thermo-oxidation of SBS copolymer during melt-compounding at high temperatures [61,62], which could also be enhanced by peroxide-induced degradation [63].

## 4. Conclusions

This paper investigated GTR modified by different grades of SBS copolymers (three linear and one radial) and cross-linking systems (sulfur-based system and dicumyl peroxide) using rheological, physico-mechanical, and morphological measurements. The results showed that SBS L7417 characterized by the highest melt flow rate and the highest content of bonded styrene among studied SBS grades was the most promising modifier of GTR processing and showed the highest compatibility confirmed by dynamic mechanical analysis. The addition of SBS copolymers to GTR increased the thermal stability of modified GTR, which might enhance the thermo-mechanical processing of cross-linked waste tire rubber and, consequently, reduce the emission of volatile degradation products generated during thermomechanical treatment. However, this assumption should be confirmed in future studies.

It was found that the highest physico-mechanical properties were obtained for GTR modified up to 30 wt% of SBS copolymer, while a further increase of SBS copolymer content in the studied materials did not lead to significant changes. The mechanical properties of modified GTR were similar (or even better) to the performance properties of commercially available reclaimed rubbers. SEM photographs showed that the GTR modified by SBS and DCP had a smoother fracture surface and a more homogeneous morphology compared to GTR-based materials with a sulfur-based system. This indicates that free radicals formed during the decomposition of DCP allow the co-cross-linking of GTR and SBS phases, which resulted in the more efficient encapsulation of GTR by SBS copolymer.

This work showed that the functionalization of GTR by styrene-butadiene copolymers is a promising strategy for waste tire management and recycling. Further investigations in this field should focus on: (i) optimization and up-scaling of GTR modification, (ii) economic aspects related to, e.g., energy consumption or used modifiers costs; (iii) environmental issues considering volatile organic compounds emission and/or undesired smell of waste tire rubber-based products.

## Figures and Tables

**Table 1:** MFR measurement results for SBS copolymers and GTR modified by SBS copolymers.

Item	SBS L7322	SBS L7342	SBS L7417	SBS R 7382	GTR +10 wt% SBS L7322	GTR +10 wt% SBS L7417
**MFR_200 °C/5 kg_ (g/10 min)**	4.2 ± 0.1	-*	23.9 ± 0.1	-*	-**	-**
**Appearance** **of the sample after test**	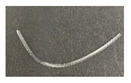	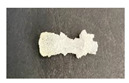	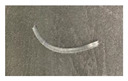	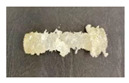	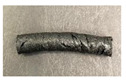	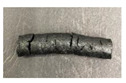
**Observations**	good flowability, transparent	poor flowability, yellowish, partial degradation	good flowability, transparent	poor flowability, yellowish, partial degradation	poor flowability	poor flowability

* unmeasurable in standardized conditions; ** unmeasurable in all measurement conditions.

**Table 2:** Mooney viscosity and curing parameters of GTR modified by SBS and cross-linking agents.

Sample Coding	Mooney Viscosity ML(1 + 4) 100 °C	M_L_ (dNm)	M_H_ (dNm)	ΔM (dNm)	t_s2_ (min)	t_90_ (min)	CRI (min^−1^)
GTR + 10 wt% SBS L7417-S	94.6	1.3	12.1	10.8	0.6	1.7	93.5
GTR + 10 wt% SBS L7417-DCP	87.1	1.5	15.0	13.5	0.9	9.7	11.4
GTR + 30 wt% SBS L7417-S	93.8	0.7	4.6	3.9	0.5	1.3	125.0
GTR + 30 wt% SBS L7417-DCP	77.5	0.9	14.3	13.5	0.8	8.7	12.7

**Table 3:** Physico-mechanical properties of GTR modified by SBS and cross-linking agents.

Sample Coding	Tensile Strength (MPa)	Elongation at Break (%)	Hardness (Shore A)	Density (g/cm^3^)
GTR + 10 wt% SBS L7417-S	7.3 ± 0.6	171 ± 15	61 ± 2	1.169 ± 0.004
GTR + 10 wt% SBS L7417-DCP	7.8 ± 0.2	190 ± 8	59 ± 1	1.133 ± 0.001
GTR + 30 wt% SBS L7417-S	7.6 ± 0.8	217 ± 23	67 ± 1	1.126 ± 0.005
GTR + 30 wt% SBS L7417-DCP	8.1 ± 0.3	202 ± 10	65 ± 2	1.077 ± 0.002

**Table 4:** Thermal decomposition characteristics of tested samples estimated from TGA data.

Sample Coding	Thermal Decomposition Temperature (°C)			
	T_−2%_	T_−5%_	T_−10%_	T_−50%_
SBS L7417	350.6	379.2	398.4	454.9
GTR	244.1	264.8	293.9	482.7
GTR + 10 wt% SBS L7417-S	255.9	314.4	365.5	458.2
GTR + 10 wt% SBS L7417-DCP	184.4	277.6	344.4	458.7
GTR + 30 wt% SBS L7417-S	253.3	317.0	365.7	457.9
GTR + 30 wt% SBS L7417-DCP	240.0	312.1	363.5	459.1

## Data Availability

Not applicable.
